# The impact of laparoscopic diverted sleeve gastrectomy with ileal transposition (DSIT) on short term diabetic medication costs

**DOI:** 10.1186/s40064-015-1216-z

**Published:** 2015-08-14

**Authors:** Alper Celik, Muharrem Asci, Bahri Onur Celik, Surendra Ugale

**Affiliations:** Metabolic Surgery Clinic, Halaskargazi Cad. Etfal Sokak Kent Pasaji No: 2/2, Sisli, Istanbul, Turkey; Bariatric and Metabolic Surgery Clinic, Kirloskar Hospital, Hyderabad, India

**Keywords:** DSIT, Surgery, Cost saving, Type 2 diabetes

## Abstract

**Background:**

Type 2 diabetes mellitus (T2DM) has gained pandemic proportions becoming a global threat within the last few decades. In parallel to the increasing prevalence, healthcare costs have become a huge economic burden for the hospital and governments. Bariatric surgery has been proven to induce glycemic control in obese type 2 diabetics. However, the cost effectiveness of metabolic surgery in overweight, obese and morbidly obese individuals has not been documented. We aimed to demonstrate the efficacy and reduced diabetic-medication cost after diverted sleeve gastrectomy with ileal interposition (DSIT) in type 2 diabetic individuals followed more than 1 year.

**Methods:**

Records of 116 type 2 diabetic patients operated by DSIT at a dedicated metabolic surgery clinic between October 2011 and April 2013 were retrospectively reviewed. A comparison was made between the annual diabetic medication cost before and after surgery using the paired t test. The alterations in BMI and HbA1c were recorded and analyzed.

**Results:**

Diverted sleeve gastrectomy with ileal interposition led to a marked reduction in BMI and improved glycemic control after 1 year follow-up. Mean HbA1c levels decreased from a mean of 8.9 ± 1.7 to 6.6 ± 1.1 1 year after surgery (p < 0.001). Mean preoperative BMI declined from 32.9 ± 4.3  to 24.7 ± 2.7 kg/m^2^ (p < 0.001). Cost of diabetic medication decreased from a mean 660.08 USD/year to 65.12 USD 1 year after surgery (p < 0.001).

**Conclusion:**

Our results have shown that DSIT operation leads to a significantly better glycemic control and lower diabetic medication costs at 1 year.

## Background

Metabolic Syndrome and especially type 2 diabetes mellitus (T2DM) became a global problem during recent decades. Its prevalence reached 8.3 % among adults and its estimated prevalence for 2030 will be 10.1 %, showing a 22 % increase (Buckley et al. 2012). According to International Diabetes Foundation (IDF) 2013 reports, Turkey has the highest prevalence of diabetes in Europe with 14.8 % of adults (International Diabetes Federation 2013). In parallel to this increasing prevalence, co-morbidities and treatment costs display a notable increase and yield a huge economic burden for insurance systems (Brennan et al. 2014). The IDF estimated that diabetes would cost the Turkish health care system 6.5b USD per year by 2030. This estimate has already been reached by 2010. The 2013 report indicates an annual diabetes related cost of 866 USD per patient (International Diabetes Federation 2013; Brennan et al. 2014; Malhan 2011; Malhan and Vlachopioti 2011).

According to national database registry of Turkey, type 2 diabetic patients constitute 95 % of all diabetics and the annual total cost of type 2 diabetes is 5.14 billion Euros. The majority of this amount is utilized for complications, whereby drug costs constitute only 10.5 % of the total expenses in type 2 diabetic patients (Malhan and Vlachopioti 2011). However, with the progression of the disease, drug usage (esp. insulin) increase and none of the currently available medications provide a solution for the continuing progressive beta cell failure (DePaula et al. 2009). Obesity surgery have shown promising and superior results with respect to metabolic control, weight control and the need for medications, by creating a reduced insulin demand and increased insulin activity (Sjöström et al. 2014; Halperin et al. 2014; Aminian et al. 2014; Rubino et al. 2009; Ghiassi et al. 2012; Chiapaikeo et al. 2014).

Currently, the most widespread procedures in obesity surgery are gastric bypass and sleeve gastrectomy. Unfortunately, the third most widespread procedures in terms of frequency are revision operations. Sadly, the word “revision” represents the disability and inefficiency of the operations performed. Just like any other field in surgery, obesity surgery also has a learning curve of its own and it is obvious that the reason of insufficient weight loss/weight regain problems occurring within the first year after surgery is technical incompetency. However, long-term follow-up results of the patients who have undergone gastric banding or sleeve gastrectomy prove that the main problem reveals itself 5 years after surgery and one-third of those patients require revision or additional surgical intervention (Chiapaikeo et al. 2014).

Diverted sleeve gastrectomy with ileal transposition (DSIT) consists of a gastric sleeve or fundectomy together with duodenal diversion and interposition of a long segment of ileum between the first centimeters of duodenum and 50th cm of jejunum. This operation has been shown to be safe and effective especially for obese and overweight patients with T2DM (DePaula et al. 2009).

The aim of the present study was to demonstrate the efficacy and reduced diabetic-medication cost after DSIT in type 2 diabetic individuals.

## Methods

### Study design

We retrospectively analyzed the prospectively collected data of 206 type 2 diabetic patients operated at a “Center of Excellence in Bariatric and Metabolic Surgery” at Taksim German Hospital between October 2011 and April 2013. All patients underwent the same surgical procedure (DSIT) carried out by the same surgical team. Of the 206 patients, 116 cases with complete 1-year postoperative follow up were enrolled in the study. Institutional Ethical Committee approval was obtained before the study was set up (14.11.2011-#11-07).

Inclusion criteria were at least 3 years duration of T2DM under stable medical treatment, HbA1c >7 % for more than 3 months, weight stability, defined as no significant change (>3 %) within the last 3 months and BMI >25 kg/m^2^. Patients were excluded if they had a fasting C peptide level <0.5 ng/ml or Anti GAD (Glutamic Adenosine De-carboxylase) Antibody positivity. Other exclusion criteria were previous major abdominal surgery, pregnancy, severe eating disorders and usage of medications for eating disorders.

### Surgical procedure

**Fig. 1:**
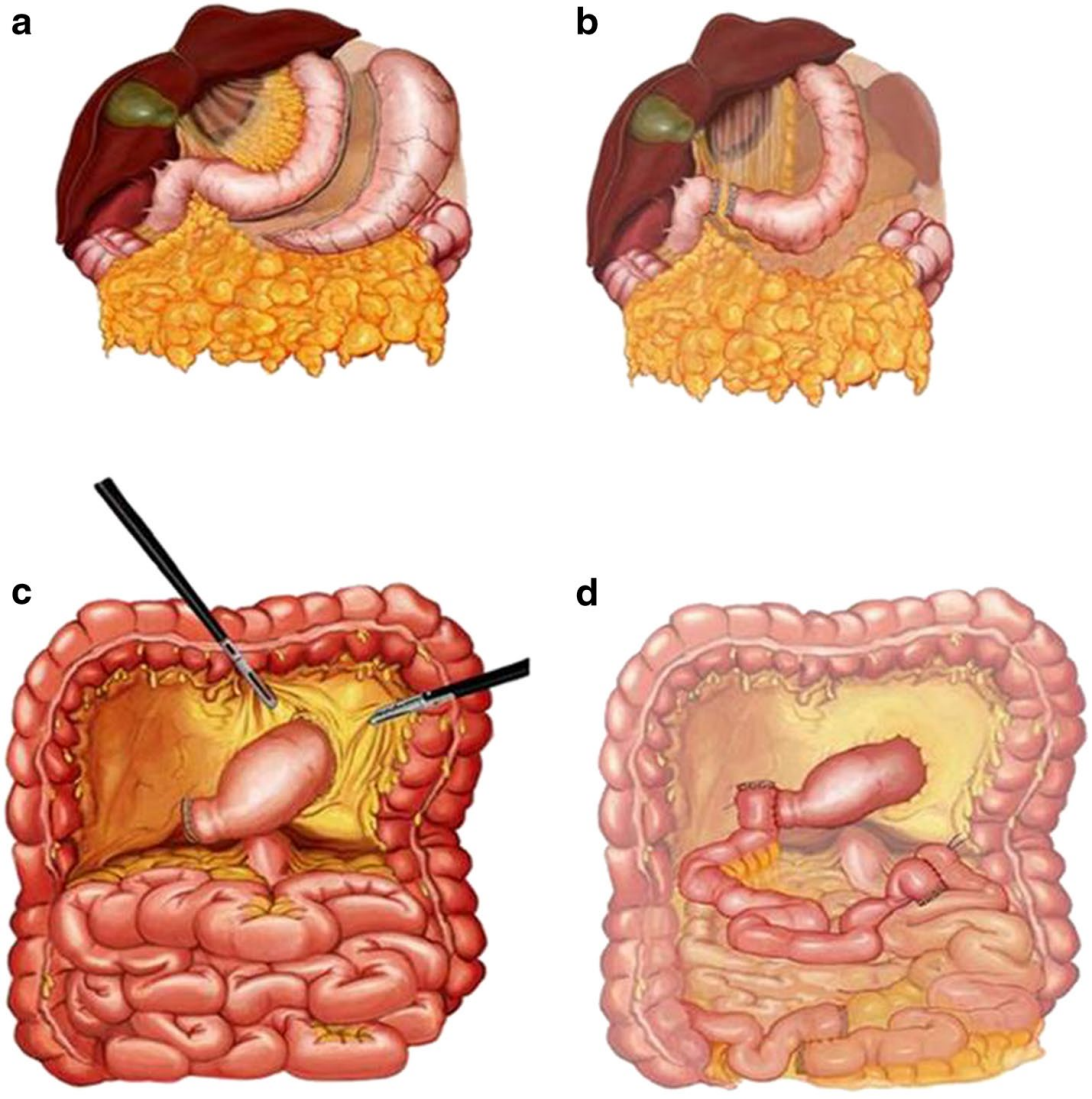
Schematic demonstration of the operation. **a **Sleeve gastrectomy.** b** Duodenal transection.** c** Inframesocolic transfer of the sleeve.** d** Interposition of the ileal segment between distal stomach and the proximal jejunum.

All patients underwent the laparoscopic DSIT operation, as previously described (Celik et al. 2014). Briefly, we start with a sleeve gastrectomy or fundectomy (depending on the BMI) and progress with duodenal transection 2–3 cm from the pylorus. This decision was based on patients’ BMI. Fundectomy was performed, leaving all antrum intact, in patients with BMI 25–30 kg/m^2^. Sleeve gastrectomy with 40F calibration plug was performed in patients with BMI 30–35 kg/m^2^. Sleeve gastrectomy with 34F calibration plug was performed in patients with BMI >35 kg/m^2^. The main rational for individual applications was not to cause weight loss more than expected due to excessive mechanical restriction. The sleeved stomach is transferred to the lower abdomen through a transverse meso-colic opening. The last 30 cm of ileum is preserved and a 170 cm segment of distal ileal segment is interposed in between the duodenal end of the stomach and 50 cm from the Treitz ligament. Ileal ends are anastomosed to each other and all the mesenteric defects are closed one by one using 3/0 polypropylene (Fig. 1).

All patients received routine multivitamin supplements for at least 6 months after surgery.

### Outcome parameters

All drug names and their doses were obtained from the BOLD (Bariatric Outcomes Longitudinal Database) system and transferred to create Excel files. The change in BMI and HbA1c were also recorded and analyzed. The drug costs were obtained from national social insurance system database and listed. Drug doses and the total daily usage was documented; costing on a daily and annual basis was evaluated accordingly. The costs have been measured in Turkish Liras (TL) and transferred to United States Dollars (USD) based on currency exchange rate of Turkish Central Bank on April 24, 2015. One USD = 2.7174 TL, http://www.tcmb.gov.tr).

### Statistical analysis

All data was transferred to IBM Statistical Package for Social Sciences (SPSS) Statistics 21.0 program for analysis (SPSS Inc., Chicago, IL, USA). The change in all dependent parameters between preoperative and postoperative 1st year was analyzed using paired *t* test. Statistical significance was set at p < 0.05.

## Results

The group of patients consisted of 79 males and 37 females with a mean age of 54.2 ± 8.7 (range 24–77) and a mean diabetic duration of 13.3 ± 5.7 (range 3–35) years. Mean preoperative BMI was 32.9 ± 4.7 (range 25.4–50.2) kg/m^2^ and mean postoperative BMI was 24.7 ± 2.7 (range 19.1–33.6) kg/m^2^. Mean change in BMI was 8.1 (range 0.7–19.2) kg/m^2^ and percentage of excess BMI loss (EBMIL) was 72.4 %. Mean weight loss at 1 year was 24.3 ± 8.1 (range −2.7 to 40.4) kg.

Mean preoperative HbA1c was 8.9 ± 1.7 which dropped down to 6.6 ± 1.1 1 year after surgery (p < 0.001). Of those, 38 patients (32.8 %) experienced a complete remission (HbA1c <6 %), and 30 patients (25.9 %) had a partial remission (HbA1c = 6–6.5 %).

Postoperatively, 26 patients (22.4 %) had an HbA1c between 6.5 and 7.5 % (2 of them were on oral antidiabetics) and 22 patients (19 %) had a 1 year HbA1c above 7.5 %. Among these 22 patients, 20 were on combination oral antidiabetics (OAD), and 2 were using OAD plus single dose of long acting insulin. By the end of 1-year follow up, 58.7 % of patients were under remission (32.8 % complete remission, and 25.9 % partial remission) without medication. 81.0 % of patients had a mean HbA1c below 7.5 %. Twenty-two patients (19 %) had an HbA1c above 7.5 %; all of whom were on insulin preoperatively, with a mean HbA1c of 9.9 ± 1.9. Mean HbA1c, of this group at 1 year was 8.5 ± 0.8 (p = 0.002).

It is of particular interest to note that a total 99 patients (83.2 %) were either on insulin only or combination of OAD with insulin preoperatively, which shows the severity of disease burden in the patient population. Preoperatively, 17 cases were on OAD, 16 were on insulin only and 83 were on insulin plus OAD. In total, 3 of 17 (17.6 %) on OAD, 3 of the 16 (18.75 %) on insulin and 16 of the 83 (19.27 %) on insulin + OAD required OAD treatment 1 year after surgery. Postoperatively, 21 % of the patients required antidiabetic treatment 1 year after surgery (Table 1).

**Table 1:** Patients pre- and postoperative antidiabetic medication requirement

Preoperative drugs	Postoperative drug use			
	None (n = 92)	Only OAD (n = 22)	OAD + insulin (n = 2)	Total (n = 116)
	n (%)	n (%)	n (%)	
Only OAD (n = 17), n (%)	14 (82 %)	3 (18 %)	0 (0 %)	(100 %)
Only insulin (n = 16), n (%)	13 (81 %)	3 (19 %)	0 (0 %)	(100 %)
OAD + insulin (n = 83), n (%)	65 (78 %)	16 (19 %)	2 (2 %)	(100 %)
Total (n = 116), n (%)	92 (79 %)	22 (19 %)	2 (2 %)	(100 %)

*OAD* oral antidiabetics.

Preoperatively, the mean cost of anti-diabetic medications was 660.08 ± 426.05 USD, which decreased to 65.12 ± 138.23 USD 1 year postoperatively (p < 0.001) (Table 2).

**Table 2:** Mean anti-diabetic medication costs in the preoperative and postoperative 1 year follow-up

Annual drug expenses (USD) (n = 116), mean ± SD		T	*p* value
Preoperative	660.08 ± 426.05	14.877	<0.001
Postoperative 1 year	65.12 ± 138.23		
Difference in expenses	594.96 ± 430.74		

*USD* United States dollars, *SD* standard deviation.

## Discussion

According to the International Diabetes Federation (IDF) report in 2013, estimated global mortality of diabetes is 5.1 million people which results in an expense of 548 billion USD (International Diabetes Federation 2013). Majority of these expenses are related to co-morbidities treatment rather than drug costs (Malhan and Vlachopioti 2011).

To the best of our knowledge, the present study is the first to analyze the effects of DSIT operation on diabetes related medication costs. Our data clearly demonstrate that surgery revealed more than 90 % reduction in the costs of anti-diabetic medications (660.08 vs. 65.12 USD, p < 0.001). Apart from costs of medication, other disease related costs such as treatment and follow-up of co-morbidities, doctor visits, blood tests and glucose measurements have not been analyzed in the present paper and may be the further investigated in other studies. We think that analysis of other disease related costs may indicate an obvious beneficial effect of surgery in all these parameters.

Within the last few decades, reports of surgical treatment have demonstrated significant benefits on T2DM and other components of metabolic syndrome (Buckley et al. 2012). However, majority of the reports belong to surgery performed on morbidly obese patients. Diverted sleeve gastrectomy with ileal transposition is a novel technique that has been shown to be safe and effective even in type 2 diabetic subjects with low BMI (Kumar et al. 2009; Kota et al. 2012). Other reports of DSIT performed on non-morbidly obese type 2 diabetic patients with a mean 39.1 (25–61) months follow up confirmed the beneficial effects and marked remission in diabetes, weight control, hypertension and dyslipidemia (DePaula et al. 2012). These effects have been documented in other publications and its effects on lipid metabolism have been particularly associated with the change in bilio-enteric circulation (Yang et al. 2013; DePaula et al. 2010). Similarly, beneficial effects on glycemia have been attributed to the improvement in insulin sensitivity and beta cell function confirmed by prospective studies (Vencio et al. 2011). Other reports of short term (18 months) follow up of non-morbidly obese patients treated by DSIT yielded 80 % remission in T2DM along with appropriate weight control without significant nutritional deficiencies. However, 20 % of patients still required treatment with OAD (Tinoco et al. 2011).

Diverted sleeve gastrectomy with ileal transposition technique is not associated with uncontrollable or severe weight loss. Postoperative weight loss is proportionate with preoperative severity of obesity. According to a study by De Paula et al. normal weight, overweight and obese patients treated by DSIT have lost a mean of 9.4, 16.8 and 23.2 kg, respectively. Irrespective of weight loss, all groups presented marked improvements in insulin output, insulin sensitivity and beta cell function (De Paula et al. 2011a, b).

All the cited articles in this manuscript indicate the efficacy of DSIT operation in controlling all the parameters linked with metabolic syndrome in a wide BMI range. Diverted sleeve gastrectomy with ileal transposition provides high rates of remission for all co-morbidities not only in morbidly obese individuals, but also in normal weight, overweight and obese patients. In the present paper, all patients were either overweight or morbid obese and there were not any patients with normal weight. Mean HbA1c dropped from a mean of 8.9 ± 1.7 to 6.6 ± 1.1 1 year after surgery. According to Turkish Ministry of Health Diabetes Prevention and Control Program, the targeted level of HbA1c should be below 6.5 % (Ministry of Health of Turkey: Turkey diabetes prevention and control program 2011). In our study, 58.7 % of our patients have achieved an HbA1c below 6.5% and 81 % of the whole patient group reached an HbA1c below 7.5 %. Only two of these patients were still on OAD, while 79 % of our patients gave up all anti-diabetic medications. Similar to our results, recent publications indicate that surgical modalities offer a more effective mode of treatment for correction of metabolic parameters in T2DM (Cotugno et al. 2015; Schauer et al. 2012).

The main limitation of our study include the retrospective design. Secondly, some details of history and factors that may influence the outcome may not be completely documented. For example, we did not take into account the cost of surgery, hospital stay, clinical follow-up of the surgical group (consultations, exams, hospitalizations), complications, safety of the procedure and the nutritional supplements that these patients should take for life. Thirdly, the present population may not be comparable to the more obese population. Finally, follow-up could only maintained in 56 % of the cases. The low follow-up rate may be due to the low socioeconomic condition, transportation difficulties and tendency of the patients not to return for follow-up once their symptoms are relieved. Due to these restrictions, associations should be interpreted with caution.

In conclusion, DSIT operation can provide lower diabetic medication costs at 1 year. Moreover, an effective weight control in a wide BMI range can be achieved. However, further prospective controlled trials are warranted to confirm these results.
